# Agreement between self-reported pre-pregnancy weight and measured first-trimester weight in Brazilian women

**DOI:** 10.1186/s12884-020-03354-4

**Published:** 2020-11-26

**Authors:** Thaís Rangel Bousquet Carrilho, Kathleen M. Rasmussen, Dayana Rodrigues Farias, Nathalia Cristina Freitas Costa, Mônica Araújo Batalha, Michael E. Reichenheim, Eric O. Ohuma, Jennifer A. Hutcheon, Gilberto Kac, Brazilian Maternal and Child Nutrition Consortium, Adauto Emmerich Oliveira, Ana Paula Esteves-Pereira, Ana Paula Sayuri Sato, Antônio Augusto Moura da Silva, Claudia Leite de Moraes, Claudia Saunders, Cristina Maria Garcia de Lima Parada, Daniela da Rocha, Denise Petrucci Gigante, Edson Theodoro dos Santos-Neto, Elisa Maria de Lacerda, Elizabeth Fujimori, Fernanda Garanhani Surita, Isabel Oliveira Bierhals, Jane de Capelli, José Guilherme Cecatti, Juliana dos Vaz, Juraci Almeida Cesar, Marco Fábio Mastroeni, Maria Antonieta de Carvalhaes, Mariângela Freitas da Silveira, Marlos Rodrigues Domingues, Mayra Pacheco Fernandes, Michele Drehmer, Mylena Maciel Gonzalez, Patrícia de Padilha, Renato Teixeira Souza, Ronaldo Fernandes Santos Alves, Rosângela Fernandes Lucena Batista, Silmara Salete de Mastroeni, Silvia Regina Dias Medici Saldiva, Simone Seixas da Cruz, Sirlei Siani Morais, Sotero Serrate Mengue

**Affiliations:** 1 Nutritional Epidemiology Observatory, Josué de Castro Nutrition Institute, Federal University of Rio de Janeiro. Avenida Carlos Chagas Filho 373/CCS, bloco J, 2 andar, sala 29. Cidade Universitária, Ilha do Fundão, Rio de Janeiro, RJ 21941-902 Brazil; 2 Division of Nutritional Sciences, Cornell University, 227 Savage Hall, Ithaca, NY 14850 USA; 3 Department of Epidemiology, Institute of Social Medicine, Rio de Janeiro State University, Rua São Francisco Xavier, 524, 7 andar, Bloco D, Sala 7018, Maracanã, Rio de Janeiro, RJ 20550-013 Brazil; 4 Maternal, Adolescent, Reproductive & Child Health (MARCH) Centre, London School of Hygiene & Tropical Medicine, Keppel Street, London, WC1E 7HT UK; 5 Centre for Tropical Medicine and Global Health, Nuffield Department of Medicine, University of Oxford, Peter Medawar Building for Pathogen Research (PMB), South Parks Road, Oxford, OX1 3SY UK; 6 Department of Obstetrics and Gynaecology, University of British Columbia, Faculty of Medicine, Suite 930, 1125 Howe Street, Vancouver, BC V6Z 2K8 Canada

**Keywords:** Weight, Self-report, First trimester, Pregnancy, Gestational weight gain, Body mass index, Agreement

## Abstract

**Background:**

Self-reported pre-pregnancy weight and weight measured in the first trimester are both used to estimate pre-pregnancy body mass index (BMI) and gestational weight gain (GWG) but there is limited information on how they compare, especially in low- and middle-income countries, where access to a weight scale can be limited. Thus, the main goal of this study was to evaluate the agreement between self-reported pre-pregnancy weight and weight measured during the first trimester of pregnancy among Brazilian women so as to assess whether self-reported pre-pregnancy weight is reliable and can be used for calculation of BMI and GWG.

**Methods:**

Data from the Brazilian Maternal and Child Nutrition Consortium (BMCNC, *n* = 5563) and the National Food and Nutritional Surveillance System (SISVAN, *n* = 393,095) were used to evaluate the agreement between self-reported pre-pregnancy weight and weights measured in three overlapping intervals (30–94, 30–60 and 30–45 days of pregnancy) and their impact in BMI classification. We calculated intraclass correlation and Lin’s concordance coefficients, constructed Bland and Altman plots, and determined Kappa coefficient for the categories of BMI.

**Results:**

The mean of the differences between self-reported and measured weights was < 2 kg during the three intervals examined for BMCNC (1.42, 1.39 and 1.56 kg) and about 1 kg for SISVAN (1.0, 1.1 and 1.2 kg). Intraclass correlation and Lin’s coefficient were > 0.90 for both datasets in all time intervals. Bland and Altman plots showed that the majority of the difference laid in the ±2 kg interval and that the differences did not vary according to measured first-trimester BMI. Kappa coefficient values were > 0.80 for both datasets at all intervals. Using self-reported pre-pregnancy or measured weight would change, in total, the classification of BMI in 15.9, 13.5, and 12.2% of women in the BMCNC and 12.1, 10.7, and 10.2% in the SISVAN, at 30–94, 30–60 and 30–45 days, respectively.

**Conclusion:**

In Brazil, self-reported pre-pregnancy weight can be used for calculation of BMI and GWG when an early measurement of weight during pregnancy is not available. These results are especially important in a country where the majority of woman do not initiate prenatal care early in pregnancy.

**Supplementary Information:**

**Supplementary information** accompanies this paper at 10.1186/s12884-020-03354-4.

## Background

Pre-pregnancy weight refers to a woman’s weight at conception and is used during prenatal care for determining maternal pre-pregnancy body mass index (BMI) and calculating gestational weight gain (GWG) [1, 2]. However, this information is not generally available in most settings because it is often not feasible to obtain weight measurements right after conception. This difficulty can be related to a variety of factors, such as unplanned pregnancies, the lack data on women’s weight throughout the life-course, and the lack of an early enough start to prenatal care for an accurate recall of women’s pre-pregnancy weight. As a result, either self-reported pre-pregnancy or measured weight in the first trimester may be used for calculating pre-pregnancy BMI and GWG [2, 3].

The agreement between self-reported and measured weight before or during the first trimester of pregnancy has been evaluated in several populations with conflicting results [4–7]. Bodnar et al. [4] evaluated the accuracy of reported pre-pregnancy BMI and GWG on over 47,000 birth certificates from Pennsylvania and found that maternal weight data was poorly reported. In contrast, Holland et al. [6] observed that using self-reported pre-pregnancy or measured weight at first prenatal visit had no impact on BMI classification using data from 307 women from Massachusetts. Some studies have also shown that maternal education, socioeconomic status, and race/ethnicity influence the quality of the reported weight [5, 8–10].

In their recent systematic review, Headen et al. [11] suggested that more studies, with larger sample sizes, are necessary to evaluate the agreement between self-reported and measured first-trimester weight, especially in the beginning of this period. They also argued that the bias in self-reporting would be problematic, especially in low- and middle-income countries, where access to a weight scale at home and medical care may be limited.

In most studies that have compared self-reported pre-pregnancy weight with weight measured in the first trimester, a restricted time frame for the measurement (such as the first month of pregnancy) was not specified. This makes it difficult to differentiate between an error in reporting pre-pregnancy weight and the possibility of weight gain during the first trimester. Three studies on this subject [12–14] have been conducted in Brazil. Oliveira et al. [12] analyzed data from 30 women from Rio de Janeiro and compared self-reported weight and the weight measured in the first trimester registered in their pregnancy cards. The authors concluded that these women underestimated their pre-pregnancy weight but, in general, the values were close to the measured weight. Niquini et al. [13] evaluated 512 women who were also from Rio de Janeiro. They compared self-reported with measured first-trimester weight and concluded that women tended to underestimate their weight but the effect of this underestimation on BMI classification was limited. Araújo et al. [14] observed the same pattern of underestimation of self-reported pre-pregnancy weight in their study of 17,093 pregnant women from the whole country. It is important to mention, however, that the agreement between self-reported pre-pregnancy weight and weight at different times during the first trimester of pregnancy was not evaluated in those studies. Thus, the possibility of using self-reported pre-pregnancy weight to determine BMI and GWG remains unclear.

The primary goal of this study was to evaluate the agreement between self-reported pre-pregnancy weight and weight measured during the first trimester of pregnancy among Brazilian women. Our primary interest was to assess whether self-reported weight was a viable option to calculate BMI and GWG in comparison with measured weight. To accomplish this, we used two large datasets, one based on research studies and another on administrative data. The secondary goal was to assess the impact of using each of these estimates in the classification of pre-pregnancy BMI.

## Methods

This study used data from two different sources, namely a research dataset from the Brazilian Maternal and Child Nutrition Consortium (BMCNC) [15], and an administrative dataset from the National Food and Nutritional Surveillance System (SISVAN, from the Portuguese acronym). The analyses were restricted to apparently healthy women (no indication of infectious or chronic diseases – except obesity - before pregnancy), aged 18–49 years.

The steps for harmonization and combination of datasets for the BMCNC are described elsewhere [15]. The harmonized and cleaned dataset included 17,344 women. The current study considered only those women who provided self-reported pre-pregnancy weight and had their weight measured in the first trimester (*n* = 5563).

The SISVAN is a national database containing nutritional surveillance information from subjects from all over the country, in every stage of life, collected during routine public health care services. It is the only Brazilian administrative dataset with repeated measures of weight during pregnancy. SISVAN had over five million women registered from 2008 to 2018. Details about the system are published elsewhere [16]. For this dataset, steps of data cleaning were employed. Identification of outliers considering the longitudinal characteristics of the data and the general distribution of variables was also performed [17, 18]. For this study, data from 393,095 women were used.

The variables used in this study included self-reported pre-pregnancy weight (in kg) collected in the first pregnancy visit or the first study interview; weight measured during the first trimester of pregnancy; maternal height (in meters) measured in the study or routine prenatal care in early pregnancy; maternal body mass index (BMI, in kg/m^2^) classified according to the World Health Organization cutoffs as underweight (< 18.5 kg/m^2^), normal (18.5–24.9 kg/m^2^), overweight (≥ 25.0 and < 30.0 kg/m^2^) and obese (≥ 30.0 kg/m^2^) [19]; and gestational age (in weeks) at the prenatal visits. The determination of gestational age varied according to the origin of the data. For the BMCNC, ultrasound data (performed before 24 weeks) and the date of the last menstrual period (LMP) reported by the women were available. Ultrasound information (date and gestational age of the exam) performed before 24 weeks was used for the determination of gestational age whenever available. When ultrasound was performed after 24 weeks or was not available, the calculation was made based on the LMP date [20]. In the SISVAN, only the date of the LMP was available.

The analyses were conducted using weight measured at several overlapping intervals during the first trimester of pregnancy, namely at 30–94 days of pregnancy (any time in the first trimester), 30–60 days (between 4 and 8 weeks), and 30–45 days (between 4 and 6 weeks). We used these intervals for both datasets as well as a cumulative analysis because we wanted to compare different potential upper gestational age ranges that clinicians and researchers might need to use. We also considered the expected pattern of weight gain up to 8th week of pregnancy, which could be as low as no gain [21]. The lower limit of 30 days was used because it is unlikely that women would start prenatal care before then. The upper limit of 94 days marks the end of the first trimester. Some women contributed more than one weight measurement during the first trimester, and, in these cases, the first measurement was used. Some women contributed to more than one group because analyses were cumulative and, thus, overlapped (e.g. if a woman had data at 42 days, these data would also be included in the other two longer intervals).

### Statistical analyses

A complete-case analysis was performed for both datasets. We determined the intraclass correlation coefficient (ICC) and Lin’s concordance coefficient, both with 95% confidence intervals (CI) [22]. ICC may vary from 0 (no agreement) to 1 (perfect agreement) and, in the current case, it assessed the agreement between the pairs of weights, representing the proportion of the total variability in the observations due to the differences between the pairs of weights [23]. Lin’s concordance coefficient can be better explained by plotting the line of best fit in a scatter plot of both weights. This coefficient is a modification of Pearson’s correlation coefficient, measuring how far the data are from the 45-degree line of perfect agreement (regression line with intercept = 0 and slope = 1). Lin’s coefficient will be equal to 1 when all the points lie on the perfect agreement line and diminishes as the points depart from this line [22].

As an initial procedure to test for the equivalence between self-reported and measured weight, two one-sided tests (TOST) were performed. The application of TOST in agreement analysis provides a means to evaluate empirical evidence of equivalence between the two measurements rather than only measuring the absence of differences, as the *t* test provides [24, 25]. To perform TOST, it is necessary to determine the equivalence region, i.e., the limits outside which the difference in mean values is considered significant (both in terms of statistics and practical use). We determined the equivalence region between − 2 and + 2 kg, considering these limits as small and plausible when evaluating the two measurements, and the upper limit of the recommended GWG for the first trimester (2 kg) [2]. We presented 90% CI since TOST consists of two separate one-sided tests at level 1-α. When combining those tests into a single CI, a confidence level of 100(1–2α)% should be estimated [24, 25].

Bland and Altman plots were also constructed [26]. These graphs portray differences between self-reported and first trimester weights, and whether these increase with higher mean weights. In perfect agreement, differences are expected to be zero irrespective of weight. Besides plotting the traditional limits at ±1.96 standard deviation proposed by the authors, we added lines indicating differences at ±2 kg to facilitate visual inspection. Bland and Altman plots were presented with different colors for each first-trimester measured BMI category, in order to show BMI variations in the differences, due to the limited sample size to perform the graphs stratified, especially in the 4–6 weeks interval.

The kappa coefficient with quadratic weighting [27] was estimated to assess the impact of using the self-reported or measured data to classify women’s pre-pregnancy BMI values. Bootstrap method with 500 replications was used to calculate 95% CI [28]. The kappa coefficient measures the agreement between two categorical variables by examining the proportion of responses in two or more agreement cells (e.g. underweight according to self-reported weight BMI/underweight according to first-trimester weight BMI) in relation to the proportion of responses in these cells which would be expected by chance, given the marginal distribution [27, 29]. The classification of agreement used the limits proposed by Landis & Koch [30]: κ > 0.60–0.80, substantial and κ > 0.80, almost perfect agreement.

As an additional analysis, to ensure that the selected women from both datasets presented similar sociodemographic profile when compared to the original data before selection, tables with means and standard deviations for continuous variables (maternal age, pre-pregnancy weight, height, pre-pregnancy BMI) and absolute and relative frequencies for categorical ones (maternal education and BMI classification) were constructed. All the analyses were performed in Stata version 15 [31] and R version 3.6 [32].

## Results

In the BMCNC, at 30–94 days, 5563 women were evaluated, at 30–60 days, 1691, and at 30–45 days, 502. In the SISVAN, the initial dataset for women with both self-reported pre-pregnancy and first-trimester measured weight was 393,095; for 30–60 days, 173,676, and for 30–45 days, 63,117 women (Additional Fig. 1). In general, women from both datasets presented similar age distributions (mean = 27.2 [SD: 5.7] years, for the BMCNC and mean = 26.8 [SD: 5.9] years for the SISVAN). However, the distribution of education was quite different among the two data sources; 17.4% of the women of the BMCNC presented tertiary education, while only 2.4% of the women in the SISVAN were in this category (Additional Table 1).

**Fig. 1:**
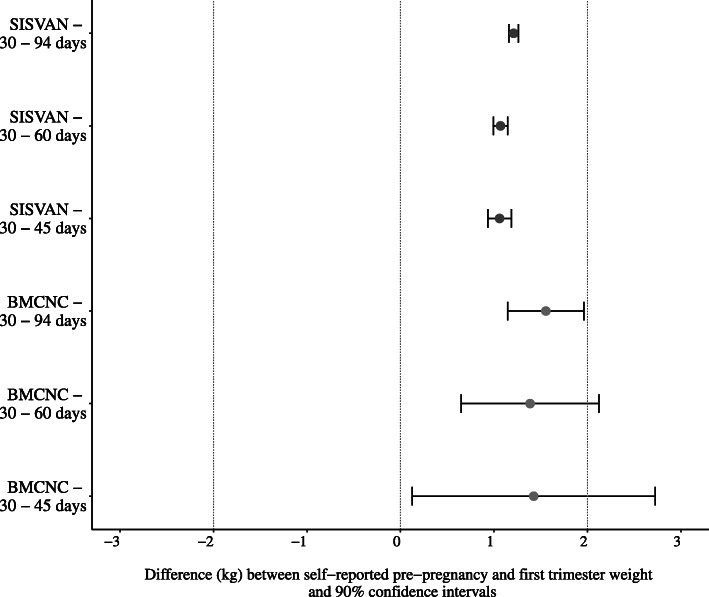
Mean differences and 90% confidence intervals for the TOST procedure for self-reported weight and weight measured in different time intervals during first trimester in the SISVAN and in the BMCNC data

In the BMCNC dataset, the mean pre-pregnancy weight was 62.4 kg (SD: 13.0), and in the SISVAN dataset, the mean was 63.8 kg (13.6). The mean first-trimester weight (measured in any gestational week from 30 to 94 days) was 64.0 (13.1) and 65.0 (13.8) kg, in the BMCNC and SISVAN, respectively. The mean of the differences between the self-reported and measured weights was less than 2 kg during the three intervals examined for the BMCNC data. These differences were smaller (around 1 kg) in the SISVAN data (Table 1).

**Table 1:** Description of the weight and body mass index (BMI) variables in the Brazilian Maternal and Child Nutrition Consortium (BMCNC) and in the Brazilian Food and Nutritional Surveillance System (SISVAN)

	BMCNC dataset		SISVAN dataset	
Weight and BMI variables	Mean (SD)	Minimum; Maximum	Mean (SD)	Minimum; Maximum
**30–94 days**				
Self-reported pre-pregnancy weight	62.4 (13.0)	30; 120	63.8 (13.6)	33.0; 128.0
First trimester weight	64.0 (13.1)	34.9; 116.8	65.0 (13.8)	36.6; 121.5
Difference between weights^a^	1.6 (3.8)	−21.1; 23.5	1.2 (2.8)	−8.7; 15.8
Self-reported pre-pregnancy BMI^b^	24.4 (4.6)	12.9; 46.9	25.0 (5.0)	12.6; 56.6
First trimester BMI^b^	25.1 (4.6)	14.4; 45.8	25.4 (5.0)	12.6; 56.9
Difference between BMI^a^	0.6 (1.48)	−7.1 – 8.7	−0.5 (1.1)	− 0.9; 4.1
**30–60 days**				
Self-reported pre-pregnancy weight	63.2 (12.9)	30; 115	64.2 (13.5)	33.0; 126.0
First trimester weight	64.6 (13.1)	35.8; 113.8	65.3 (13.7)	36.6; 120.0
Difference between weights^a^	1.4 (3.5)	−21.1; 19.5	1.1 (2.6)	−8.7; 15.8
Self-reported pre-pregnancy BMI^b^	24.6 (4.6)	13.9; 45.7	25.0 (4.9)	12.6; 53.0
First trimester BMI^b^	25.1 (4.7)	14.4; 45.8	25.4 (5.0)	12.6; 53.3
Difference between BMI^a^	0.5 (1.3)	−7.1 – 8.0	−0.4 (1.0)	−7.4; 4.1
**30–45 days**				
Self-reported pre-pregnancy weight	64.2 (12.5)	40; 112	64.6 (13.7)	33.0; 124.0
First trimester weight	65.6 (12.5)	36; 113.8	65.6 (13.7)	36.6; 119.0
Difference between weights^a^	1.4 (3.2)	−16.0; 18.0	1.0 (2.5)	−8.7; 15.8
Self-reported pre-pregnancy BMI^b^	24.9 (4.5)	16.4; 40.4	25.1 (4.9)	12.6; 53.0
First trimester BMI^b^	25.5 (4.5)	16.0; 40.2	25.5 (5.0)	12.6; 53.3
Difference between BMI^a^	0.6 (1.3)	−5.5 – 7.8	−0.4 (1.0)	−7.4; 4.0

Notes: ^a^ Difference was calculated as: first trimester weight/BMI – self-reported weight/BMI. ^b^ There is a small difference in the sample size for the calculation of BMI due to missing data in height in the BMCNC dataset (n = 5381 for 30–94 days, n = 1617 for 30–60 days, n = 480 for 30–45 days). *SISVAN* Brazilian Food and Nutritional Surveillance System, *BMCNC* Brazilian Maternal and Child Nutrition Consortium, *SD* Standard deviation

ICC and Lin’s coefficient were above 0.90 for both datasets. Higher agreement was observed between self-reported and the weight measured between 30 and 45 days of pregnancy (ICC and Lin = 0.961 and 0.979 for the BMCNC data and the SISVAN, respectively), compared to the other time intervals. All the coefficients were slightly smaller when the first-trimester weight measured at any time point (30–94 days) was used, compared to more restricted time intervals (30–60 and 30–45 days) (Table 2).

**Table 2:** Measures of agreement between self-reported weight and weight measured during the first pregnancy trimester among Brazilian women in the Brazilian Maternal and Child Nutrition Consortium (BMCNC) and in the Brazilian Food and Nutritional Surveillance System (SISVAN)

	30–94 days (up to 13 weeks)	30–60 days (up to 8 weeks)^a^	30–45 days (up to 6 weeks)^a^
**BMCNC**			
Number of individuals	5563	1691	502
ICC (95% CI)	0.952 (0.949–0.954)	0.959 (0.956–0.963)	0.961 (0.954–0.967)
Lin’s coefficient (95% CI)	0.952 (0.949–0.954)	0.959 (0.956–0.963)	0.961 (0.953–0.967)
Kappa coefficient (95% CI^b^)	0.867 (0.857–0.875)	0.888 (0.868–0.903)	0.897 (0.865–0.923)
Mean gestational age in the period (days)	69	49	40
**SISVAN**			
Number of individuals	393,095	173,676	63,117
ICC (95% CI)	0.976 (0.975–0.976)	0.978 (0.978–0.979	0.979 (0.979–0.980)
Lin’s coefficient (95% CI)	0.976 (0.975–0.976)	0.978 (0.978–0.979	0.979 (0.979–0.980)
Kappa coefficient (95% CI^b^)	0.909 (0.909–0.910)	0.918 (0.917–0.920)	0.923 (0.921–0.925)
Mean gestational age in the period (days)	63.3	47.9	39.8

Notes: ^a^Cumulative analysis. ^b^500 replications; there is a small difference in the sample size for the calculation of the Kappa coefficient for BMI due to missing data in height, in the BMCNC dataset (*n* = 5381 for 30–94 days, *n* = 1617 for 30–60 days, *n* = 480 for 30–45 days). *BMCNC* Brazilian Maternal and Child Nutrition Consortium, *CI* Confidence interval, *ICC* Intraclass correlation coefficient, *SD* Standard deviation, *SISVAN* Brazilian Food and Nutritional Surveillance System

The TOST procedure revealed that self-reported pre-pregnancy weight and first trimester measures can be considered equivalent, but not equal, for all the evaluated scenarios in the SISVAN but only for 30–94 days in the BMCNC data, according to 90% CI and ± 2 kg limits used. For the BMCNC data, the 90% CIs for the periods of 30–45 days and 30–60 days were outside those limits, so these measurements cannot be considered equivalent according to this procedure (Fig. 1**)**.

**Fig. 2:**
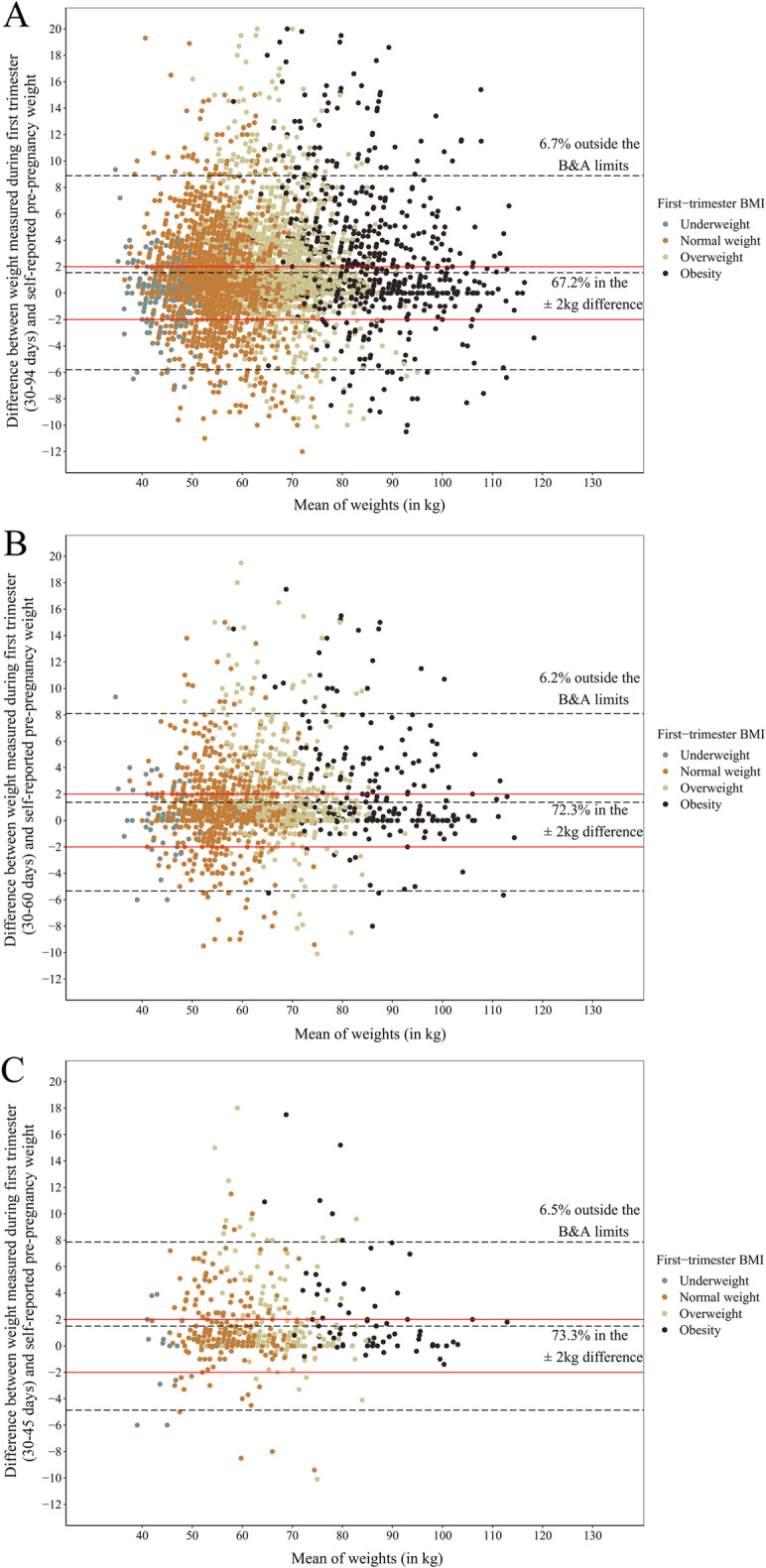
Bland and Altman plots for the agreement between self-reported pre-pregnancy weight and weight measured in the BMCNC data: **a** 30–94 days of pregnancy; **b** 30–60 days of pregnancy; **c** 30–45 days of pregnancy. Note: 1. **b** & **a** limits: Limits as defined in Bland & Altman (1986): Mean difference ± 1.96 X Standard deviation of the difference. 2. A small number of individuals (*n* = 18 in A, *n* = 4 in **b** and n = 1 in **c**) were not plotted due to the chosen limits of the plots. 3. There is a small difference in the sample size in the graphs due to missing data in height (*n* = 5381 for 30–94 days, *n* = 1617 for 30–60 days, *n* = 480 for 30–45 days)

**Fig. 3:**
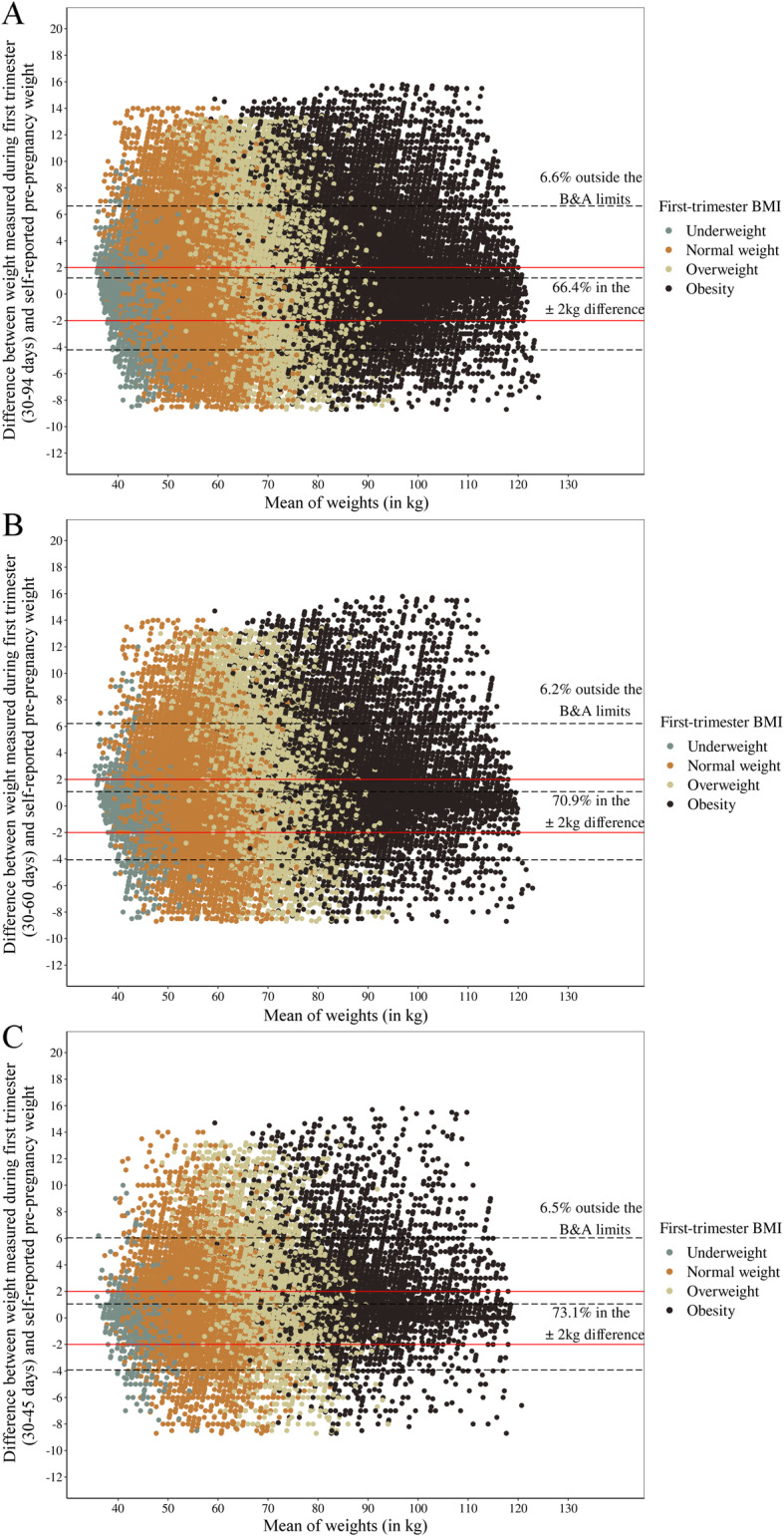
Bland and Altman plots for the agreement between self-reported pre-pregnancy weight and weight measured in the SISVAN data: **a** 30–94 days of pregnancy; **b** 30–60 days of pregnancy; **c** 30–45 days of pregnancy. Note: **b** & **a** limits: Limits as defined in Bland & Altman (1986): Mean difference ± 1.96 X Standard deviation of the difference

The Bland and Altman plots (Figs. 2 and 3) showed that most women were within the limits of agreement defined by the plot (93.3, 93.6, 93.2% and 93.4, 93.8, 93.5% for the BMCNC and SISVAN at 30–94, 30–60, and 30–45 days, respectively). The percentage of women within the ±2 kg difference between the weights varied between 66 and 73% according to the time interval studied and the dataset used. Similarly, the percentage of women with differences above 5 kg is low (~ 10%, data not shown). The plots indicated no differing pattern regarding the mean of weights, i.e., the differences are spread over the distribution of means of self-reported and first-trimester weight in both datasets for all periods. It was also not possible to observe different patterns regarding the classification of first-trimester BMI.

Kappa coefficient varied from substantial to almost perfect agreement. Slightly higher values (0.89 for the BMCNC data and 0.92 for the SISVAN) occurred when the BMI was classified using the first-trimester weight measured up to 30–45 days, but the results were similar in other periods, especially if the CI is taken into account (Table 2). The high values of this coefficient highlight the low proportions off the agreement cells, showing that most women are classified in the same BMI category when self-reported or first-trimester weight was used (Tables 3 and 4).

**Table 3:** Contingency tables for the classification of body mass index (BMI) using self-reported and first trimester weight in the Brazilian Maternal and Child Nutrition Consortium (BMCNC)

Self-reported pre-pregnancy weight	First trimester weight				
	Underweight	Normal	Overweight	Obesity	Total
**30–94 days**					
Underweight	**167 (3.1)**	121 (2.2)	0	0	288 (5.3)
Normal	41 (0.8)	**2597 (48.3)**	393 (7.3)	6 (0.1)	3037 (56.4)
Overweight	0	103 (1.9)	**1148 (21.3)**	153 (2.8)	1404 (26.1)
Obesity	0	0	41 (0.8)	**611 (11.3)**	652 (12.1)
Total	208 (3.9)	2821 (52.4)	1583 (29.4)	770 (14.3)	5381 (100)
**30–60 days**					
Underweight	**53 (3.3)**	29 (1.8)	0	0	82 (5.1)
Normal	7 (0.4)	**792 (49.0)**	107 (6.6)	1 (0.06)	907 (56.1)
Overweight	0	27 (1.7)	**361 (22.3)**	40 (2.5)	428 (26.5)
Obesity	0	0	7 (0.4)	**193 (11.9)**	200 (12.4)
Total	60 (3.7)	848 (52.4)	475 (29.4)	234 (14.5)	1617 (100)
**30–45 days**					
Underweight	**14 (2.9)**	4 (0.8)	0	0	18 (3.7)
Normal	3 (0.6)	**225 (46.9)**	34 (7.1)	1 (0.2)	263 (54.8)
Overweight	0	4 (0.8)	**116 (24.2)**	13 (2.7)	133 (27.7)
Obesity	0	0	0	**66 (13.7)**	66 (13.7)
Total	17 (3.5)	233 (48.5)	150 (31.2)	80 (16.7)	480 (100)

Note: Values refer to absolute and relative frequencies in each cell. BMI cutoffs: Underweight, BMI < 18.5 kg/m^2^; Normal, BMI ≥ 18.5 and < 25.0 kg/m^2^; Overweight, BMI ≥ 25.0 and < 30.0 kg/m^2^ and Obesity, BMI ≥ 30.0 kg/m^2^

**Table 4:** Contingency tables for the classification of body mass index (BMI) using self-reported and first trimester weight in the Brazilian Food and Nutritional Surveillance System (SISVAN)

Self-reported pre-pregnancy weight	First trimester weight				
	Underweight	Normal	Overweight	Obesity	Total
**30–94 days**					
Underweight	**13,351 (3.4)**	7195 (1.8)	0	0	20,546 (5.2)
Normal	2342 (0.6)	**185,445 (47.2)**	21,083 (5.4)	17 (< 0.001)	208,887 (53.1)
Overweight	0	4619 (1.2)	**88,762 (22.6)**	9844 (2.5)	103,265 (26.3)
Obesity	0	0	2290 (0.6)	**58,107 (14.8)**	60,397 (15.4)
Total	15,693 (4.0)	197,259 (50.2)	112,135 (28.5)	68,008 (17.3)	393,095 (100)
**30–60 days**					
Underweight	**5819 (3.4)**	2658 (1.5)	0	0	8477 (4.9)
Normal	951 (0.5)	**82,339 (47,4)**	8373 (4.8)	8 (< 0.001)	91,671 (52.8)
Overweight	0	1824 (1.1)	**40,779 (23.5)**	4049 (2.3)	46,625 (26.9)
Obesity	0	0	904 (0.5)	**25,972 (14.9)**	26,876 (15.5)
Total	6770 (3,9)	86,821 (50.0)	50,056 (28.8)	30,029 (17.3)	173,676 (100)
**30–45 days**					
Underweight	**1984 (3.1)**	934 (1.5)	0	0	2918 (4.6)
Normal	308 (0.5)	**29,901 (47.4)**	2946 (4.7)	2 (< 0.001)	33,157 (52.5)
Overweight	0	602 (0.9)	**15,114 (23.9)**	1387 (2.2)	17,103 (27.1)
Obesity	0	0	279 (0.4)	**9660 (15.3)**	9939 (15.7)
Total	2292 (3.6)	31,437 (49.8)	18,339 (29.1)	11,049 (17.5)	63,117 (100)

Note: Values refer to absolute and relative frequencies in each cell. BMI cutoffs: Underweight, BMI < 18.5 kg/m^2^; Normal, BMI ≥ 18.5 and < 25.0 kg/m^2^; Overweight, BMI ≥ 25.0 and < 30.0 kg/m^2^ and Obesity, BMI ≥ 30.0 kg/m^2^

Using self-reported pre-pregnancy or measured weight would change, in total, the classification of BMI in 15.9, 13.5, and 12.2% of women in the BMCNC at 30–94, 30–60, and 30–45 days, respectively. For the SISVAN, the reclassification would be 12.1, 10.7, and 10.2% for the same periods. The larger discrepancy was observed for the classification at 30–94 days for both datasets. In this interval, for the BMCNC, 7.3% of the women would be classified as normal using self-reported and as overweight if the first-trimester weight were used (Table 3). In the SISVAN, 5.4% of women would be classified as normal with self-reported weight and overweight with first-trimester weight. Most women changed their BMI classification to an adjacent category and larger discrepancies in classification were not observed in either dataset (Table 4).

## Discussion

We observed a substantial agreement (all coefficients had values > 0.80) between self-reported weight and the weight measured in the first trimester, especially if the latter was measured up to the first 30–45 days of pregnancy. The weight measurement in this period, closer to conception, would be the best choice in research and clinical practice to determine BMI and GWG when recovering the true pre-conceptional weight is not possible, because it is less likely to be affected by weight gain. However, a measurement in this restricted time frame is rarely available in most settings. Thus, self-reported pre-pregnancy weight seems to be a suitable choice.

As we expanded the length of the eligible first-trimester window, the difference between the two measures was somehow larger, which would be expected, as there is usually weight gain along the first trimester. Weight measurements collected during the first trimester in a larger time frame (30–60 or 30–94 days) are also commonly used for the calculation of BMI and GWG. Nevertheless, using those measurements for calculating GWG would be neglecting pregnancy weight variations during this period, which is expected to happen [21]. In turn, the acceptable agreement observed with these other time intervals reinforces the possibility of using self-reported weight in both research and clinical practice. This finding corroborates with Park et al. [33] results, using birth certificate data from the United States, which observed minimal and not clinically meaningful differences between pre-pregnancy and measured first-trimester weight.

Most studies on this topic have collected the information of self-reported weight in different times relative to the last menstrual period, i.e., in the beginning, middle or at the end of the gestational period or even 10 years after the conception [34, 35]. This may lead to important recall bias given the long period since the event occurred. In addition, few studies have compared the report to the weight actually measured in the first trimester and, even if they did, the comparison was only one to point in the first trimester of pregnancy [8, 36].

In their systematic review, Headen et al. [11] observed a high correlation between self-reported and early-pregnancy weight, despite a tendency toward underreporting of pre-pregnancy weight among the 33 identified studies. In the three studies conducted in Brazil, a high correlation and agreement between self-reported and measured weight and a tendency toward underestimation was also observed [12–14]. However, these studies did not consider the timing of the first-trimester weight measurement and the possibility of weight gain/loss during this period. These factors could produce differences between the report and the measurements that would not result from misreporting.

The TOST showed that for the SISVAN and the BMCNC data for 30–94 days, the weights could be considered statistically equivalent. These results emphasize that there is a difference between the measures, but this difference is expected, mainly because the first-trimester weight will be higher than pre-pregnancy weight. This would occur even if women remember this value correctly. In terms of magnitude of discrepancies, the means were around 2 kg for both datasets and all time intervals, and in the Bland and Altman plots, the majority of the difference (~ 66–77%) were found in the ±2 kg interval. Shin et al. [8] also observed that self-reported weight was lower than measured first-trimester weight by an average of 2.3 kg in data from 504 pregnant women from the United States. The 2-kg difference is close to the expected weight gain in the first trimester [2] and may not be related to an inaccurate weight report, but to rather to real changes in the measures because of the expected weight gain during this interval. Also, the plots revealed no pattern of difference considering measured first-trimester BMI, which may indicate that the BMI classification does not change the way a woman report her pre-pregnancy weight.

The classification of pre-pregnancy BMI using self-reported or first-trimester measured weight also had a high agreement, i.e., the impact of using one weight or another in the classification of BMI was small, according to the values of the kappa coefficient. The reduced impact on the classification suggests that using self-reported rather than measured weight would not substantially bias BMI estimates. This result differs from the findings of Fattah et al. [36]. These authors evaluated 100 women from Ireland and observed that 22% of the women were classified in different BMI categories whether self-reported or measured first-trimester weight was used in BMI calculation.

For ascertainment of GWG, the differences observed between the weights would be the same for weight gain calculated using self-reported pre-pregnancy or measures first-trimester weight. However, since measurements collected early in the first trimester are rarely available, using weight collected at any time during this period would not consider the possibility of weight gain during the first trimester. Maternal first-trimester weight change may be important to fetal growth and the child’s future health [37–39]. In addition, using first-trimester weight to calculate GWG would be problematic for those women who start prenatal care after this time frame.

Recently published GWG charts by Santos et al. [40] and Hutcheon et al. [41] used self-reported weight to calculate weight gain and classify pre-pregnancy BMI, considering self-reported weight as the most commonly available information. However, the Intergrowth-21st GWG [42] and the Swedish charts [43] used weight measured in the first trimester, considering it a more appropriate source for the creation of the charts. It is possible to observe that there is still debate as to which measure should be employed, especially in studies assessing GWG [11, 44]. However, considering the substantial agreement observed in the current study, it seems reasonable to assume the majority of women would receive adequate GWG counseling based on self-reported information.

By using a time interval close to conception (30–45 days), we were able to show that the women’s report was not substantially biased and could be used when a measurement of weight in the beginning of pregnancy is unavailable. We also argue that using a measurement of weight collected at any time during the first trimester, although more often available, might disregard an effective GWG in this period. Future studies with weight measurements of women before conception could focus on developing a correction factor or a calibration equation to obtain a more accurate value for ascertaining both pre-pregnancy BMI and GWG. This would only be possible if weighting non-pregnant women becomes part of their routine health care.

### Strengths and limitations

The availability of both self-reported and measured weights in the first trimester on the same women permitted us to address the central question of this study. Besides, using the most available realistic approaches for first-trimester weight also allowed us to evaluate differences when each weight was used. Collecting self-reported weight at the beginning of pregnancy to help reducing recall bias is another important strength. Also, the large sample size in both datasets for the first-trimester measurement used in the analyses and the fact that these datasets are from a middle-income country, where only limited information on this subject has been available, must be highlighted.

Nevertheless, some limitations must be considered. The number of women with a weight measurement in the first 30–45 and 30–60 days was relatively low, which affected the comparison of the coefficients and the determination of the TOST CI, even though the sample sizes for these periods were higher than those observed in several studies previously conducted on the topic [5, 35, 44]. The sample size was not big enough to estimate coefficients according to BMI category, which would have been useful to compare differences between the report according to those categories. However, Bland and Altman plots were constructed depicting categories of BMI with different colors, and no clear pattern of differences were observed.

The reduction in the sample size in both datasets is another limitation to be mentioned. Unfortunately, the proportion of women who initiate prenatal care before 13 weeks in Brazil is limited and selecting those who also know their pre-pregnancy weight contributed further to decreasing the sample size available for this study in both sources of data. These facts may raise concern about the profile of the selected women, who may be substantially different from those removed from the analysis. However, the comparison of sociodemographic characteristics between all the women and those selected for this study revealed a remarkably close profile (Additional Table 1).

The use of data collected in the routine prenatal care, which was the case for most studies included in the BMCNC and the SISVAN, could also introduce noise to the evaluation of the agreement between the measurements because the collection of weight and height was not standardized. The precise question used to inquire women about their pre-pregnancy weight is unknown. In some studies, participants knew their weight; in other cases, this information was abstracted from the participants’ pregnancy booklets/cards. Finally, it was not possible to know the timing of the collection of self-reported weight, i.e., if it occurred before or after women were weighted in the visit. If women reported their pre-pregnancy weight shortly after being weighted, knowing the weight could have influenced their report. We recommend that the timing of weight is considered in future studies in the field.

## Conclusions

In this study, we observed substantial agreement between self-reported pre-pregnancy and first-trimester measured weight, mainly at the beginning of gestation (30–45 days), even in data from SISVAN, a national administrative system. The mean differences between self-reported and measured first-trimester weights were lower than 2 kg, which have little impact on BMI classification and GWG calculation. An early measurement of weight is rarely available in Brazil and other LMIC and self-reported pre-pregnancy weight can be easily collected or retrieved from medical records or pregnancy booklets/cards. Thus, it seems to be a suitable choice to estimate pre-pregnancy BMI and GWG among Brazilian women and also those from other countries with similar characteristics.

## Supplementary Information

**Additional file 1: Table S1.** Comparison between all women and those selected for the analyses (with information on self-reported pre-pregnancy weight and first-trimester measured weight) in the Brazilian Maternal and Child Nutrition Consortium (BMCNC) and National Food and Nutritional Surveillance System (SISVAN) data.

**Additional file 2: Figure S1.** Flowchart for the constitution of the datasets used in this study: **a**. data from the Brazilian Maternal and Child Nutrition Consortium (BMCNC), and **b**. the National Food and Nutritional Surveillance System (SISVAN).

## Data Availability

The data that support the findings of this study are available from the Brazilian Maternal and Child Nutrition Consortium, but restrictions apply to the availability of these data, which were used under license for the current study, and so are not publicly available. Data are however available from the authors upon reasonable request and with permission of all members of the Consortium. SISVAN data is available upon request to the Ministry of Health.

## Abbreviations

BMCNC: Brazilian Maternal and Child Nutrition Consortium
BMI: Body mass index
CI: Confidence interval
GWG: Gestational weight gain
IOM: Institute of Medicine
LMP: Last menstrual period
SISVAN: National Food and Nutritional Surveillance System
TOST: Two one-sided tests
