# Supplementary material for: Agreement between self-reported pre-pregnancy weight and measured first-trimester weight in Brazilian women
Source: BMC Pregnancy Childbirth. 2020 Nov 26;20:734. doi: 10.1186/s12884-020-03354-4 (PMC7690094; doi:10.1186/s12884-020-03354-4)
Supplement: Supplementary file 1 — Additional file 1: Table S1. Comparison between all women and those selected for the analyses (with information on self-reported pre-pregnancy weight and first-trimester measured weight) in the Brazilian Maternal and Child Nutrition Consortium (BMCNC) and National Food and Nutritional Surveillance System (SISVAN) data. [file 12884_2020_3354_MOESM1_ESM.docx]

**Additional table 1. Comparison between all women and those selected for the analyses (with information on self-reported pre-pregnancy weight and first-trimester measured weight) in the Brazilian Maternal and Child Nutrition Consortium (BMCNC) and National Food and Nutritional Surveillance System (SISVAN) data.**

|  | BMCNC | | SISVAN | |
| --- | --- | --- | --- | --- |
|  | All women  (n=17,344) | Selected women  (n=5563) | All women  (n= 840,848) | Selected women (n=393,095) |
| Continuous variables | Mean (SD) | Mean (SD) | Mean (SD) | Mean (SD) |
| Age | 26.9 (5.8) | 27.2 (5.7) | 26.7 (5.9) | 26.8 (5.9) |
| Pre-pregnancy weight (kg) | 60.8 (13.1) ^b^ | 62.4 (13.0) | 63.4 (13.4) | 63.8 (13.6) |
| Maternal height (cm) | 159.3 (7.0) ^c^ | 159.7 (7.0) | 159.5 (6.4) | 159.8 (6.4) |
| Pre-pregnancy BMI | 24.1 (4.6) ^d^ | 24.4 (4.6) | 24.9 (4.9) | 24.9 (5.0) |
|  |  |  |  |  |
| Categorical variables | n (%) | n (%) | n (%) | n (%) |
| Maternal education ^a^ |  |  |  |  |
| Pre-primary | 1775 (10.8) | 557 (10.0) | 116,001 (25.5) | 48,337 (21.1) |
| Primary | 4447 (27.0) | 1385 (25.0) | 288,194 (63.2) | 150,648 (65.7) |
| Secondary | 7895 (47.9) | 2644 (47.6) | 42,468 (9.3) | 24,734 (10.8) |
| Tertiary | 2358 (13.3) | 963 (17.4) | 8967 (2.0) | 5461 (2.4) |
|  |  |  |  |  |
| Pre-pregnancy BMI |  |  |  |  |
| Underweight | 814 (6.1) | 288 (5.4) | 47,103 (5.6) | 20,517 (5.2) |
| Normal weight | 7915 (59.6) | 3037 (56.4) | 452,005 (53.8) | 207,819 (52.9) |
| Overweight | 3101 (23.4) | 1404 (26.1) | 219,105 (26.0) | 104,164 (26.5) |
| Obesity | 1442 (10.9) | 652 (12.1) | 122,635 (14.6) | 60,595 (15.4) |

Notes: ^a^ Classification according to ISCED 2011 Operational Manual: Guidelines for Classifying National Education Programmes and Related Qualification; ^b^ available for 13,765 women; ^c^ available for 16,448 women; ^d^ available for 13,272 women.
