# Supplementary material for: Agreement between self-reported pre-pregnancy weight and measured first-trimester weight in Brazilian women
Source: BMC Pregnancy Childbirth. 2020 Nov 26;20:734. doi: 10.1186/s12884-020-03354-4 (PMC7690094; doi:10.1186/s12884-020-03354-4)
Supplement: Supplementary file 2 — Additional file 2: Figure S1. Flowchart for the constitution of the datasets used in this study: a. data from the Brazilian Maternal and Child Nutrition Consortium (BMCNC), and b. the National Food and Nutritional Surveillance System (SISVAN). [file 12884_2020_3354_MOESM2_ESM.pdf]

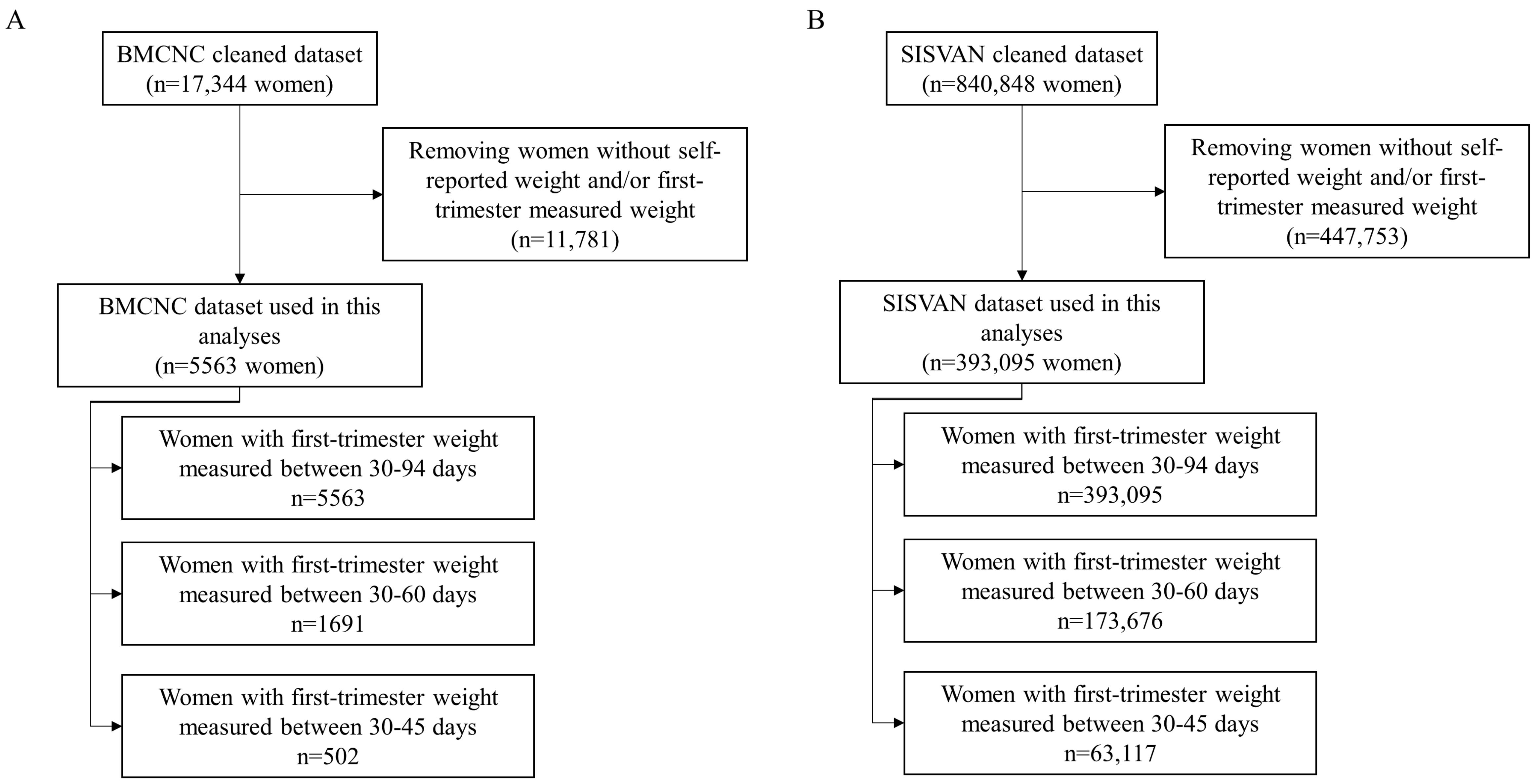


**Additional figure 1.** Flowchart for the constitution of the datasets used in this study: **a.** data from the Brazilian Maternal and Child Nutrition Consortium (BMCNC), and **b.** the National Food and Nutritional Surveillance System (SISVAN).
